# Genome-Wide Association Study and Candidate Gene Analysis of Seed Shattering Trait in *Psathyrostachys juncea*

**DOI:** 10.3390/genes16111383

**Published:** 2025-11-14

**Authors:** Yuru Lv, Lan Yun, Yixin Mu, Bohua Li, Xiaodi Jia, Miaomiao Jia

**Affiliations:** 1 College of Grassland Science, Inner Mongolia Agricultural University, Hohhot 010018, China; yurulv@163.com (Y.L.); muyixin@emails.imau.edu.cn (Y.M.); m17614915013@163.com (B.L.); jiaixaodi@163.com (X.J.); 13847317933@163.com (M.J.); 2 Research Institute of Biotechnology, Inner Mongolia Academy of Agricultural and Animal Husbandry Sciences, Hohhot 010031, China

**Keywords:** *Psathyrostachys juncea*, seed shattering, GWAS, candidate gene, *CESA*

## Abstract

Background: Seed shattering enhances ecological adaptation in perennial grasses but severely limits harvestable seed yield in forage crops. *Psathyrostachys juncea* is an important perennial forage species in arid and cold regions, yet the genetic basis of its seed shattering remains largely unknown. Here we asked which genomic regions and biological pathways underlie natural variation in seed shattering in *P. juncea*, and whether cellulose synthase (*CESA*)-mediated cell-wall formation contributes to abscission-zone strength. Results: We evaluated seed shattering in a diverse association panel of *P. juncea* across four environment–-year combinations and performed a genome-wide association study (GWAS) using genotyping-by-sequencing single-nucleotide polymorphism (SNP) markers. The analysis identified 36 significant SNP loci distributed on multiple chromosomes, consistent with a highly polygenic and environment-responsive architecture. Candidate-gene annotation highlighted pathways related to cell-wall biosynthesis, hormone signaling and sugar transport. Notably, in the BT23SHT environment a cluster of association signals on chromosome 3D co-localized with several genes annotated as cellulose synthase (*CESA*). Abscission-zone transcriptome profiling and qRT-PCR at 7, 14, 21 and 28 days after heading revealed that *CESA* genes, including *TraesCS3D02G010100.1* located near the lead SNP Chr3D_3539055, showed higher early expression in low-shattering lines and a decline toward baseline in high-shattering lines. Comparative analyses placed *P. juncea CESA* proteins within a broadly conserved but lineage-divergent framework among grasses. Conclusion: Together, these results define the genetic landscape of seed shattering in *P. juncea* and nominate cellulose-biosynthetic genes on chromosome 3D as promising targets for marker-assisted selection of low-shattering, high-seed-yield forage cultivars.

## 1. Introduction

*Psathyrostachys juncea*, commonly known as Russian wildrye, is a vital perennial grass species in the Poaceae family. It is widely distributed across the arid and semi-arid regions of central and northern Asia, where it plays an important role in stabilizing soils, improving grassland ecosystem functions and supporting livestock production. This species is particularly valued for its remarkable drought tolerance, salinity resistance and ability to control wind erosion, making it a critical resource for grassland restoration and grazing management, especially in northern China. Moreover, *P. juncea* exhibits early regrowth, prolonged greenness and strong grazing tolerance, together with high nutritional value in autumn, thereby integrating forage production, ecological restoration and economic benefits [1].

Despite these advantages, the extensive utilization of *P. juncea* is severely hindered by excessive seed shattering, which leads to substantial yield losses at maturation. Seed shattering directly reduces seed retention on the panicle and lowers the efficiency of forage seed production. As a consequence, seed yield remains a bottleneck for the large-scale application of *P. juncea*, particularly in grassland rehabilitation and forage crop production [1,2]. Seeds are essential for grass reproduction, and their yield is a key determinant of the success of establishment, ecological restoration and rehabilitation of degraded grasslands. In China, the demand for forage seeds has far outpaced domestic production, resulting in a growing reliance on imports [3]. This situation highlights an urgent need for innovative breeding programs that enhance seed yield and seed retention traits such as seed shattering, with clear agronomic, ecological and economic implications for modern grassland industries.

Seed shattering, a critical trait in many grass species, arises from complex genetic and physiological mechanisms involving cell-wall composition, mechanical strength of the abscission zone and hormone signaling pathways [4]. In natural ecosystems, seed shattering is adaptive because it promotes seed dispersal and population persistence. However, in agricultural and restoration contexts, where high seed retention is essential to maximize harvestable yield and reduce seeding costs, excessive shattering is undesirable [4]. Understanding the genetic basis of seed shattering in *P. juncea* is therefore critical for developing effective breeding strategies aimed at improving seed yield and stand establishment. Nevertheless, this trait is typically quantitative, polygenic and strongly influenced by the environment, which has so far limited progress in dissecting its underlying genetic control [5,6,7,8].

In response to these challenges, genome-wide association studies (GWAS) have emerged as a powerful approach for uncovering the genetic architecture of complex traits in natural or breeding populations. By correlating phenotypic data with high-density single nucleotide polymorphism (SNP) markers, GWAS enables the identification of genomic loci controlling quantitative traits with relatively high resolution [9,10,11,12]. In recent years, GWAS has made significant contributions to crop improvement by clarifying the genetic basis of key agronomic traits such as salinity tolerance in wheat [13], drought resistance in maize [14], cold tolerance in rice [15], insect resistance in cotton [16] and rust resistance in maize [17]. In forage crops, GWAS has also been applied, although its use is still emerging. For example, a recent study in alfalfa uncovered multiple loci associated with forage quality [18], and studies in *Setaria italica* and *Lolium multiflorum* identified QTLs and loci related to shattering or shattering-associated regions [19,20]. However, compared with major cereals, research on seed traits-particularly seed shattering-in perennial forage grasses remains underexplored, and *P. juncea* still lacks a systematic genetic dissection of this trait.

Recent studies have increasingly focused on integrating transcriptomic data with GWAS to improve the resolution of genetic mapping and to identify functional genes directly involved in important traits [21,22,23,24]. Such integrative approaches are especially valuable for complex traits like seed shattering, where multiple biological pathways and strong environmental modulation are involved. However, for *P. juncea* there is still a clear gap in connecting genomic regions associated with seed shattering to expression variation in the abscission zone and to specific candidate genes that can be exploited in breeding.

Therefore, in this study we combined multi-environment phenotyping, GWAS and transcriptomic analyses to investigate the genetic foundation of seed shattering in *P. juncea*. Specifically, we aimed to: (i) characterize phenotypic variation in seed shattering in a diverse *P. juncea* association panel evaluated across four environment-year combinations; (ii) identify SNP loci associated with seed shattering using a GWAS framework based on genotyping-by-sequencing markers; (iii) integrate GWAS signals with abscission-zone RNA-seq and qRT-PCR data to prioritize candidate genes related to cell-wall biosynthesis and abscission-zone development; and (iv) perform comparative and phylogenetic analyses of cellulose synthase (*CESA*) genes to place *P. juncea CESA* in a broader evolutionary context. In brief, our workflow proceeded from multi-environment phenotypic evaluation and SNP genotyping, through genome-wide association mapping and candidate-gene annotation, to expression profiling and comparative analyses of *CESA* genes, with the ultimate goal of generating practical genetic targets for improving seed yield and retention in *P. juncea* and other perennial Poaceae forage crops.

## 2. Results

### 2.1. Data Distribution Characteristics and Visualization

**Figure 1:**
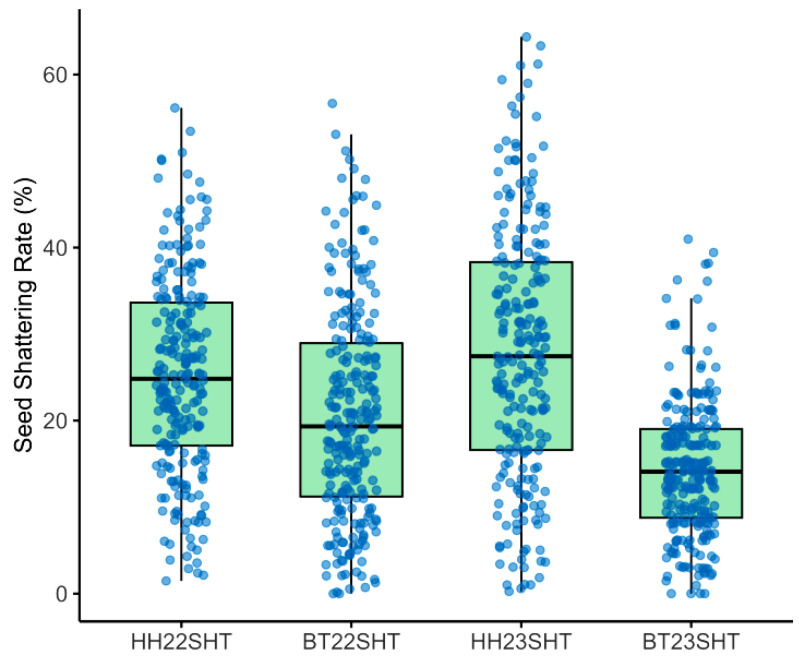
Boxplots of seed shattering rates. HH22SHT (Hohhot 2022), BT22SHT (Baotou 2022), HH23SHT (Hohhot 2023), BT23SHT (Baotou 2023).

**Figure 2:**
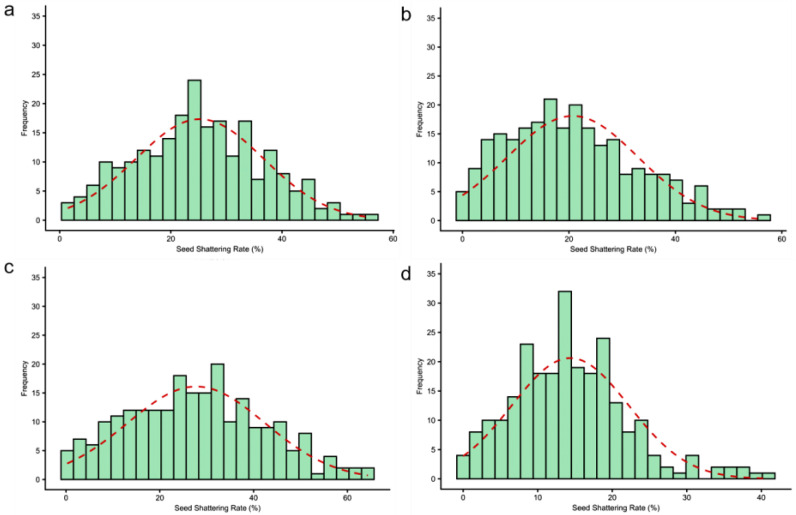
Histogram of seed shattering rates. (**a**) HH22SHT (Hohhot 2022), (**b**) BT22SHT (Baotou 2022), (**c**) HH23SHT (Hohhot 2023), (**d**) BT23SHT (Baotou 2023). The red dashed line represents the fitted normal distribution curve based on the sample mean and standard deviation.

**Figure 3:**
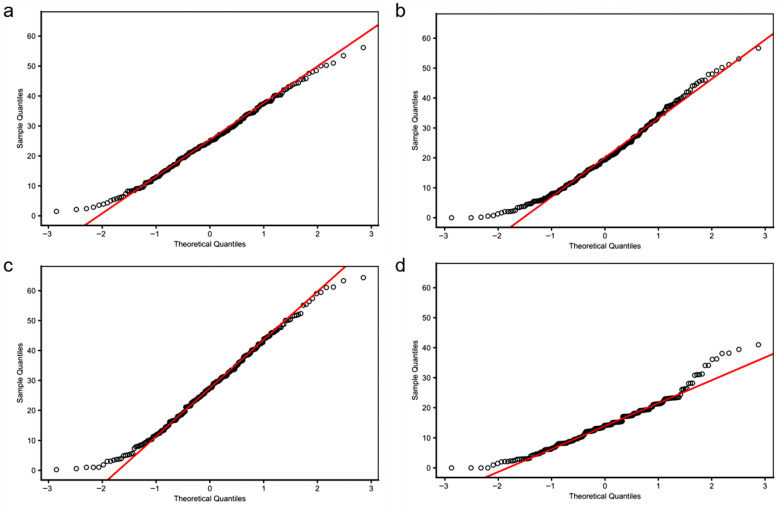
QQ image of seed shattering rates. (**a**) HH22SHT (Hohhot 2022), (**b**) BT22SHT (Baotou 2022), (**c**) HH23SHT (Hohhot 2023), (**d**) BT23SHT (Baotou 2023).

To investigate the environmental effects on seed shattering, we analyzed seed shattering rates across four environment-year combinations: HH22SHT (Hohhot 2022), BT22SHT (Baotou 2022), HH23SHT (Hohhot 2023), and BT23SHT (Baotou 2023). Figure 1 shows the boxplots of seed shattering rates across the four environments. The mean seed shattering rates were 25.21%, 20.70%, 27.76%, and 14.37% for HH22SHT, BT22SHT, HH23SHT, and BT23SHT, respectively. HH22SHT and HH23SHT exhibited broader phenotypic ranges (0–56.15% and 0–64.35%), indicating greater variability, while BT23SHT showed a narrower range (0–40.98%). Figure 2 presents frequency histograms with overlaid normal distribution curves. The distributions of HH22SHT (Figure 2a) and HH23SHT (Figure 2c) were approximately symmetric, suggesting near-normality, while BT22SHT (Figure 2b) and BT23SHT (Figure 2d) were right-skewed, indicating deviations from normality. This suggests that environmental factors in Baotou may have constrained shattering trait variation. Figure 3 shows Q-Q plots for normality assessment. Data from HH22SHT and HH23SHT closely followed the theoretical quantile line, supporting normality, while BT22SHT and BT23SHT showed clear tail deviations, confirming non-normality in these datasets.

### 2.2. Normality Test

The normality of the seed shattering rates for HH22SHT, BT22SHT, HH23SHT, and BT23SHT was assessed using the Shapiro–Wilk (SW) and Kolmogorov–Smirnov (KS) tests [25,26], with results summarized in Table 1. The SW test, which was the primary method in this study due to its higher sensitivity, revealed that HH22SHT had a *p*-value of 0.212, indicating no significant deviation from normality. HH23SHT had a borderline *p*-value of 0.021, suggesting slight deviation. In contrast, BT22SHT and BT23SHT showed significant deviations from normality with *p*-values of 0.0007 and 0.001, respectively. Although the KS test produced higher *p*-values (e.g., 0.964 for HH22SHT, 0.591 for BT22SHT, and 0.246 for BT23SHT), it is less sensitive, particularly for smaller sample sizes, and may underestimate non-normality. Based on the SW test results, BT22SHT and BT23SHT are considered non-normally distributed. The discrepancy between the two tests is noteworthy. The SW test detected non-normality in BT22SHT and BT23SHT due to its sensitivity to skewness and minor deviations, while the KS test, more suited for larger datasets, indicated normality for all variables. Therefore, we selected the SW test as the primary method for normality assessment in this study due to its greater sensitivity and applicability to smaller sample sizes. A summary of the results is presented in Table 1.

### 2.3. Analysis of Manhattan and Q-Q Plots

**Figure 4:**
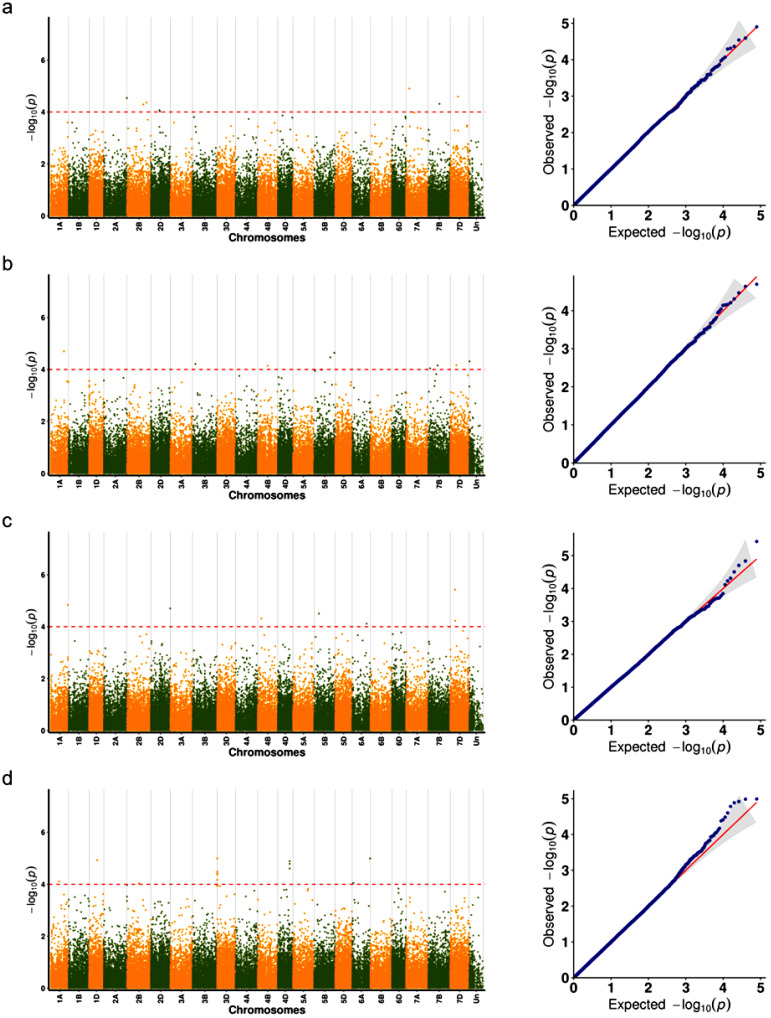
Manhattan and Q-Q plots of genome-wide association results for seed shattering under four environments. Each subfigure (**a**–**d**) represents a specific environment: (**a**) HH22SHT (Hohhot 2022), (**b**) BT22SHT (Baotou 2022), (**c**) HH23SHT (Hohhot 2023), and (**d**) BT23SHT (Baotou 2023). Manhattan plots are shown on the left and Q-Q plots on the right. The red dashed line represents the significance threshold (−log_10_(*p*) = 4).

**Figure 5:**
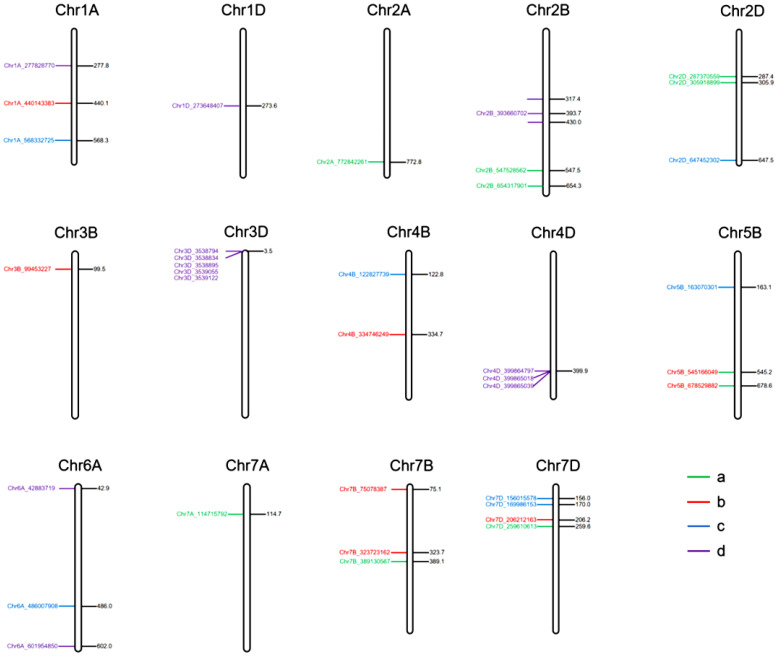
Distribution of 36 significant SNPs associated with the seed shattering trait across 14 wheat chromosomes. Panels (**a**–**d**) correspond to the four environment-year combinations: (**a**) HH22SHT (Hohhot 2022), (**b**) BT22SHT (Baotou 2022), (**c**) HH23SHT (Hohhot 2023), (**d**) BT23SHT (Baotou 2023).

Based on the GWAS results for seed shattering traits under four environments (HH22SHT, BT22SHT, HH23SHT, and BT23SHT), the distribution and statistical significance of the associated SNP loci are presented in Figure 4 and Figure 5, with Manhattan plots shown on the left and Q-Q plots on the right. In the Manhattan plots, significant SNP loci exceeding the red dashed threshold were observed in all four environments, with clear variation in the number and chromosomal distribution of associated loci. After applying Bonferroni correction, the *p*-values ranged from 0.001 to 0.005, and FDR correction resulted in adjusted *p*-values ranging from 0.003 to 0.02. For instance, in HH22SHT and HH23SHT, significant SNPs were mainly concentrated on chromosome 7D, while in BT23SHT, they were more widely distributed on chromosome 3D. No overlapping loci were identified across all four environments. The Q-Q plots showed that most observed *p*-values followed the expected distribution under the null hypothesis, with slight deviations at the upper tails. These deviations indicate the presence of potential genetic associations within individual environments. These results suggest that SNP associations detected vary across environments, and no loci were commonly significant in all cases.

### 2.4. Chromosomal Distribution of SNPs Associated with Seed Shattering Trait

In environment HH22SHT, a total of 8 significant SNP loci were identified, distributed across chromosomes 2A, 2B, 2D, 7A, 7B, and 7D, explaining 1.12% to 9.59% of the phenotypic variance. After applying Bonferroni correction, the *p*-values ranged from 0.001 to 0.005, and FDR correction resulted in adjusted *p*-values ranging from 0.003 to 0.02. Notably, the Chr7D_259610613 locus on chromosome 7D exhibited the highest contribution (9.59%), indicating its potential role as a key regulatory region associated with seed shattering traits (Table 2). In environment BT22SHT, 8 significant SNP loci were detected, distributed across chromosomes 1A, 3B, 4B, 5B, 7B, and 7D, with individual loci explaining 4.80% to 8.91% of the phenotypic variance. Among these, the Chr3B_99453227 locus on chromosome 3B demonstrated the highest contribution (8.91%) (Table 2). In environment HH23SHT, 7 significant SNP loci were identified, located on chromosomes 1A, 2D, 4B, 5B, 6A, and 7D, with phenotypic variance explained ranging from 4.31% to 8.85%. The Chr1A_568332725 locus on chromosome 1A showed the highest contribution (8.85%) (Table 2). In environment BT23SHT, 13 significant SNP loci were detected, distributed across chromosomes 1A, 1D, 2B, 3D, 4D, and 6A, explaining 5.37% to 10.58% of the phenotypic variance. The Chr6A_601954850 locus on chromosome 6A contributed the most, accounting for 10.58% of the phenotypic variance (Table 2). Across environments HH22SHT, BT22SHT, HH23SHT, and BT23SHT, a total of 36 significant SNP loci associated with seed shattering traits were identified. These loci were distributed across chromosomes 1A, 2A, 2B, 2D, 3B, 4B, 5B, 6A, 7A, 7B, and 7D, with phenotypic variance explained ranging from 1.12% to 10.58%. The distribution of these SNPs exhibited clear environmental specificity, emphasizing the environmental dependence of the genetic regulation for this trait. Chromosome 7D displayed significant SNPs in three out of the four environments, with particularly strong signals observed in environments HH22SHT and HH23SHT, indicating its potential importance in seed shattering regulation under specific environmental conditions. Additionally, chromosome 3D also showed significant SNPs in environment BT23SHT, with loci (Chr3D_3538794, Chr3D_3538834, Chr3D_3538895, Chr3D_3539055, and Chr3D_3539122) contributing to phenotypic variance ranging from 7.8% to 9.08%, suggesting a key regulatory role for chromosome 3D in environment BT23SHT. Moreover, chromosomes 1A and 3B exhibited significant signals in specific environments, such as BT22SHT and HH23SHT, implying that these chromosomes may have environment-specific regulatory functions. The minor allele frequency (MAF) of the significant SNPs ranged from 0.05 to 0.35, reflecting considerable genetic diversity within the population and providing valuable insights for the identification of key genes involved in seed shattering.

### 2.5. Candidate Gene Annotation for Seed Shattering

From the GWAS of seed shattering, a total of 28 candidate genes were identified (Table S1). These genes were associated with significant SNP loci in different environments.

In HH22SHT, 8 significant SNP loci were identified, leading to the discovery of 5 candidate genes. Notably, the gene *TraesCS2A02G580500.1* on chromosome 2A, associated with the SNP Chr2A_772842261, is annotated as a disease resistance protein (RPP13), potentially involved in stress responses. In addition, *TraesCS2B02G383800.1* (chromosome 2B), annotated as a BTB/POZ and MATH domain protein, and *TraesCS2B02G460000.1* (chromosome 2B), annotated as O-methyltransferase ZRP4, were identified near SNP loci Chr2B_547528562 and Chr2B_654317901, respectively. On chromosome 7B, *TraesCS7B02G213300.1* (glyceraldehyde-3-phosphate dehydrogenase) was associated with SNP Chr7B_389130567.

In BT22SHT, 8 significant SNP loci were identified, corresponding to 4 candidate genes. *TraesCS1A02G248200.1* (chromosome 1A), a Rhomboid family protein, was located near SNP Chr1A_440143383, and TraesCS3B02G125200.1 (chromosome 3B), annotated as 1-aminocyclopropane-1-carboxylate synthase, was associated with SNP Chr3B_99453227. On chromosome 5B, *TraesCS5B02G513600.1*, a haloacid dehalogenase hydrolase, was found near SNP Chr5B_678529882, and *TraesCS7B02G069800.1* (chromosome 7B), a receptor serine/threonine protein kinase, was associated with SNP Chr7B_75078387.

In HH23SHT, 7 significant SNP loci were identified, leading to the discovery of 9 candidate genes. Notably, *TraesCS1A02G404900.1* and *TraesCS1A02G405600.1* on chromosome 1A, associated with SNP Chr1A_568332725, were annotated as a Myb DNA-binding domain protein and a sugar transporter ERD6, respectively, which are involved in gene expression regulation and material exchange. Other key genes include *TraesCS2D02G592200.1* (F-box/LRR protein) and *TraesCS2D02G592500.1* (nucleotide hydrolase) on chromosome 2D, as well as *TraesCS5B02G110900.1* (fructose-bisphosphate aldolase) on chromosome 5B, associated with metabolic processes.

In BT23SHT, 13 significant SNP loci were identified, revealing 10 candidate genes. A major focus was on chromosome 3D, where five candidate genes annotated as cellulose synthase were identified near SNP loci Chr3D_3538794, Chr3D_3538895, Chr3D_3539055, and Chr3D_3539122, with *TraesCS3D02G010100.1* contributing significantly to seed shattering regulation. Additionally, on chromosome 4D, *TraesCS4D02G237600.1* was identified near SNP loci Chr4D_399864797, and associated with U-box domain-containing proteins. On chromosome 6A, *TraesCS6A02G074600.1* and *TraesCS6A02G382500.1* were annotated as ribosomal proteins and a bidirectional sugar transporter, SWEET13, which are involved in metabolic regulation and material transport.

These candidate genes provide valuable insights into the genetic mechanisms underlying seed shattering and offer potential targets for breeding low-shattering cultivars.

### 2.6. Haplotype Analysis of Seed Shattering

**Figure 6:**
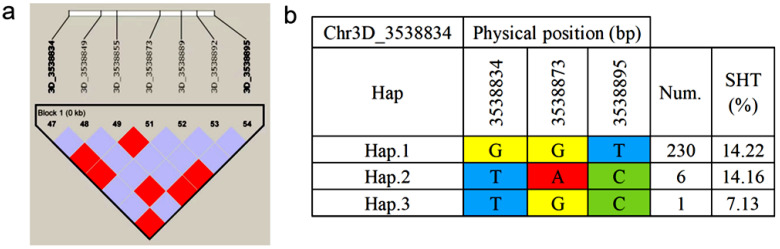
Haplotype analysis of Chr3D_3538834. (**a**) Linkage disequilibrium (LD) block structure of Chr3D_3538834 and six flanking SNP markers generated using HaploView (version 4.2); the color gradient from purple to red indicates increasing pairwise LD between markers. (**b**) Haplotypes constructed from three SNPs within the LD block, showing the nucleotide combinations (Hap.1-Hap.3), the number of individuals carrying each haplotype (Num.), and their mean seed shattering rate (SHT, %); colored cells represent nucleotides (yellow, G; red, A; blue, T; green, C).

Haplotype analysis of all associated loci for seed shattering traits in *P. juncea* was performed using HaploView software (version 4.2). Results revealed that the SNP locus Chr3D_3538834 formed a 0 kb linkage region with six markers (Figure 6a). Within this region, three haplotypes were identified (Figure 6b): haplotype-GGT (Hap.1), haplotype-TAC (Hap.2), and haplotype-TGC (Hap.3). Among the 300 *P. juncea* individuals analyzed, 230 carried Hap.1 with an average seed shattering rate of 14.22%; 6 individuals carried Hap.2 with an average seed shattering rate of 14.16%; and 1 individual carried Hap.3 with the lowest seed shattering rate of 7.13%. These findings suggest that Hap.3 is a potentially superior haplotype for improving seed retention.

### 2.7. Analysis of Key Candidate Gene Expression Levels

**Figure 7:**
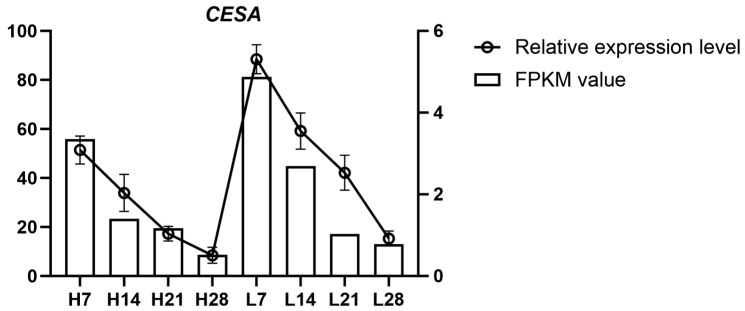
Expression analysis of the *CESA* gene in the abscission zone of *Psathyrostachys juncea*. L indicates low seed-shattering lines and H indicates high seed-shattering lines; numbers (7, 14, 21, 28) denote days after heading. Bars represent RNA-seq expression values (FPKM, left *y*-axis), and lines represent qRT-PCR relative expression levels (right *y*-axis). Dual y-axes display different measurement scales and are not directly comparable. Error bars indicate variation among biological replicates.

Among the candidate genes identified through GWAS, five were located near significant SNP loci on chromosome 3D in the BT23SHT environment, all annotated as cellulose synthase (*CESA*) genes. *CESA* is a key enzyme in cellulose biosynthesis, which is crucial for the strength and integrity of the plant cell wall. Modifications in cellulose content, especially in the abscission layer, are known to influence seed shattering in grasses. Therefore, *CESA* genes were considered strong candidates for regulating seed shattering. To examine *CESA* gene expression, transcriptome sequencing (RNA-seq) was performed on abscission zone tissues from high and low seed-shattering groups at four developmental stages (7, 14, 21, and 28 days after heading). A representative gene, *TraesCS3D02G010100.1*, located 23.6 kb from the significant SNP locus Chr3D_3539055, was selected for detailed analysis. The expression dynamics of *CESA* showed clear differences between the high shattering rate group (H group) and the low shattering rate group (L group) (Figure 7). In the H group, RNA-seq (FPKM) and qRT-PCR data consistently showed a decrease in expression from H7 to H28, with the highest expression at H7 and nearly undetectable levels at H28 (*p* < 0.05). In contrast, the L group exhibited higher *CESA* expression at all stages, with the highest expression at L7, followed by a gradual decline through L14, L21, and L28 (*p* < 0.05). Despite this decline, *CESA* expression in the L group remained higher than in the H group at each time point. These results indicate that higher *CESA* expression is associated with reduced seed shattering, while lower expression correlates with enhanced shattering.

### 2.8. Phylogenetic Analysis of CESA Genes

**Figure 8:**
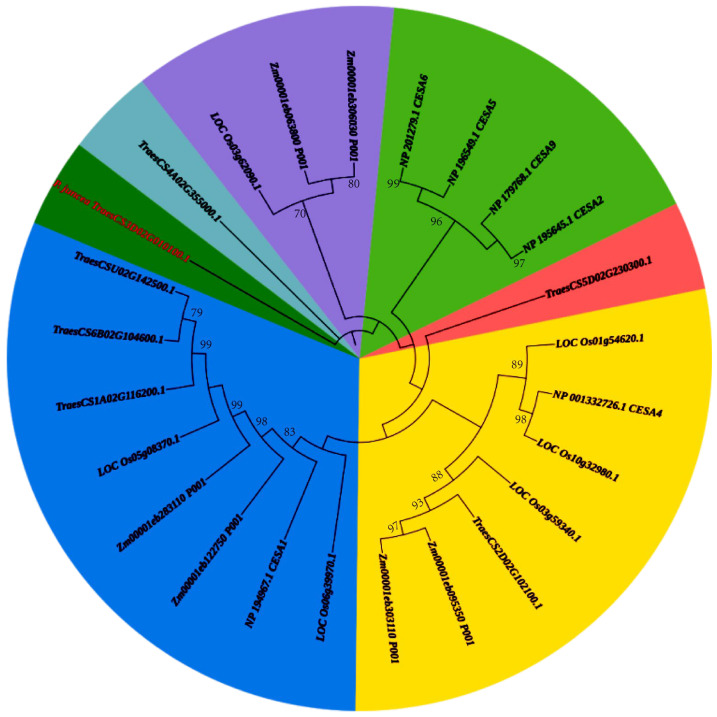
Phylogenetic analysis of *CESA* genes in *P. juncea* and related species. Different background colors represent different phylogenetic clades.

Phylogenetic analysis of *CESA* genes across five plant species—*P. juncea*, *Oryzasativa*, *Zea mays*, *Triticum. aestivum*, and *Arabidopsis thaliana*—revealed significant evolutionary divergence (Figure 8). *CESA* genes from monocot species (*O. sativa, Z. mays*, and *T. aestivum*) formed tightly clustered branches, indicating high sequence conservation and functional consistency in cellulose synthesis for primary and secondary cell walls. Within this group, *T. aestivum* exhibited additional sub-branching, suggesting functional divergence among its *CESA* family members. In contrast, *A. thaliana*, representing dicot species, formed a separate clade, reflecting the distinct evolutionary path and functional adaptation of *CESA* genes in dicots. The *CESA* gene from *P. juncea* (*TraesCS3D02G010100.1*) diverged early from the other monocots, indicating unique sequence characteristics or evolutionary trajectories. This early divergence may be associated with ecological adaptations, such as tolerance to arid or saline environments, highlighting the specialized evolutionary path of *CESA* genes in *P. juncea*. These findings underscore the evolutionary conservation and differentiation of *CESA* genes across monocots and dicots, while also emphasizing the potential functional specialization of *CESA* genes in *P. juncea*. This study lays the groundwork for further exploration of *CESA* gene functionality and its role in plant adaptation and cellulose biosynthesis.

### 2.9. Comparative Study Based on Motif Analysis

**Figure 9:**
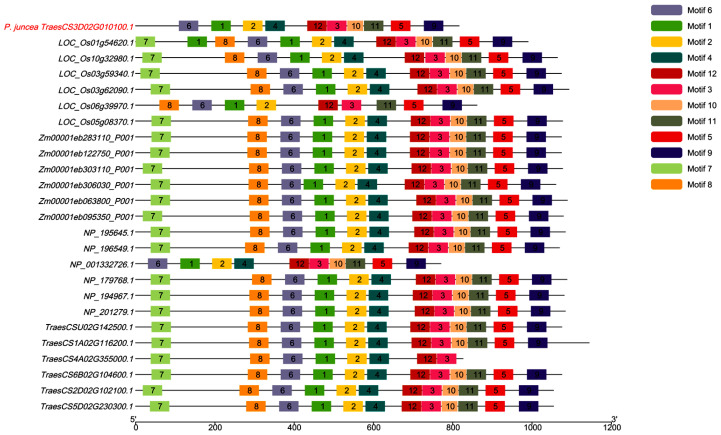
Comparative motif analysis of *CESA* genes in *P. juncea* and related species.

We analyzed the motif distribution patterns of *CESA* genes across five species: *Psathyrostachys juncea*, *Oryza sativa*, *Triticum aestivum*, *Zea mays*, and *Arabidopsis thaliana* (Figure 9). Most *CESA* genes retained all 12 motifs, though some motifs were absent or varied in distribution across species. For example, *TraesCS3D02G010100.1* in *P. juncea* lacks motifs 7 and 8, which may indicate evolutionary specialization in cellulose biosynthesis regulation. Similarly, LOC_Os05g08370.1 in *O. sativa* lacks motifs 4, 7, and 10, suggesting optimization of cellulose synthase in rice for cell wall formation. The *NP_001332726.1* gene in *A. thaliana* is missing motifs 7 and 8, while *TraesCS4A02G355000.1* in *T. aestivum* lacks motifs 5, 9, 10, and 11. These variations reflect significant functional divergence in *CESA* genes among species, pointing to adaptive changes in regulatory pathways in response to biological needs. In particular, *P. juncea* shows unique adaptive changes in cellulose biosynthesis and cell wall regulation. While it shares similarities with other monocots such as rice, maize, and wheat, it exhibits distinct differences in motif distribution. These differences may be linked to adaptations in response to extreme environments, such as drought and salinity, suggesting an ecological advantage for *P. juncea.* Further analysis revealed that motifs 1, 2, 3, 6, and 12 were highly conserved across all species, with consistent length and sequence composition. These conserved motifs likely play a central role in cellulose biosynthesis, potentially involved in catalytic activity and structural stability. In contrast, the absence of motifs 7 and 8 may alter the regulatory properties of cellulose synthases, potentially affecting protein interactions or specific metabolic pathways. Overall, these findings highlight both the evolutionary conservation and species-specific characteristics of *CESA* genes, suggesting that the missing motifs reflect adaptive changes in cellulose biosynthesis regulation. This provides valuable insights for future functional studies of *CESA* genes.

The motif analysis of *CESA* genes in *P. juncea* was performed using InterProScan, which identified 12 key motifs with distinct functional roles. Motif 1, the cellulose synthase domain (Pfam PF03552), is crucial for cellulose biosynthesis. Motif 2, associated with X-box transcription factors (PANTHER PTHR13301), likely plays a role in gene regulation. Motif 3, the zinc finger domain (SSF57850), is important for signal transduction, while Motif 4, a membrane-bound region (Phobius TMhelix), anchors *CESA* proteins to the membrane. Motif 5, an extracellular region (Phobius Non-Cytoplasmic Domain), may be involved in cellulose export, and Motif 6, a transmembrane region (TMHMM, Phobius), helps maintain membrane association. Motif 7, another cellulose synthase domain (Pfam PF03552), further supports cellulose production, while Motif 8, which includes both transmembrane and cytoplasmic regions (Phobius, TMhelix), is involved in synthesis and signaling. Motif 9, another X-box transcription factor-related motif (PANTHER PTHR13301), suggests a further regulatory role. Motif 10, a zinc finger, RING/FYVE/PHD-type domain (Gene3D), facilitates regulation via metal ion binding, while Motif 11, a nucleotide-diphospho-sugar transferase domain (Gene3D), is involved in sugar transfer for cellulose biosynthesis. Finally, Motif 12, a transmembrane region (Phobius), further supports the membrane association of *CESA*. These motifs collectively define the functional roles of *CESA* genes in cellulose biosynthesis and regulation.

## 3. Discussion

Seed shattering enables ecological adaptation and population dispersal in Poaceae but compromises seed yield and harvest stability. In this study, a GWAS across four environment-year settings identified 36 significant SNPs distributed on multiple chromosomes (including 1A, 1D, 2A, 2B, 2D, 4B, 4D, 6A, among others), each explaining 1.12–10.58% of the phenotypic variance, consistent with a polygenic and environment-responsive architecture in *Psathyrostachys juncea.* This pattern aligns with observations in *Oryza sativa*, where shattering-related QTLs were mapped on chromosomes 1, 2, 4, 6, and 7 with comparable contribution rates [27]. Because a high-quality *P. juncea* reference is not yet available, alignment to the wheat genome maximized mapping and annotation but may influence chromosomal interpretation due to genome divergence, motivating future work with *P. juncea* or closely related diploid assemblies.

Integration of association mapping with expression profiles. In the BT23SHT environment, a cluster of five significant loci on chromosome 3D was found in close physical proximity to genes annotated as cellulose synthase (*CESA*). Abscission-zone expression profiling at 7, 14, 21, and 28 days after heading revealed distinct group- and time-dependent differences between low- and high-shattering lines (Figure 7). Both RNA-seq (FPKM) and qRT-PCR showed higher early expression in low-shattering lines, whereas expression in high-shattering lines progressively declined to near baseline by 28 days. At each corresponding time point, *CESA* expression levels in the low-shattering group remained higher than those in the high-shattering group. The representative gene *TraesCS3D02G010100.1*, located 23.6 kb from the lead SNP Chr3D_3539055, exhibited the same temporal trend. These concordant association and expression results connect the BT23SHT 3D region with abscission-zone transcriptional differences relevant to shattering under that environment (Figure 7).

Seed shattering is a complex trait regulated by multiple genes that influence developmental and cell-separation processes. In HH22SHT, a WPP domain–associated protein near Chr2D_305918899 has been implicated in ABA-linked organ abscission in pepper [28], and a BTB/POZ–MATH protein near Chr2B_547528562 participates in reproductive development in rice [29]. An O-methyltransferase near Chr2B_654317901 is involved in lignin biosynthesis, a contributor to cell wall composition [30]. In BT22SHT, a receptor serine/threonine protein kinase near Chr7B_75078387 has roles related to cell separation during dehiscence in oilseed species [31]. In HH23SHT, an LRR protein and a MYB DNA-binding domain (near Chr1A_568332725) relate to pathways linked with cell wall synthesis and hormone signaling in *A. thaliana* [32]. Loci associated with fructose-bisphosphate aldolase and a nucleotide hydrolase (Chr5B_163070301; Chr2D_647452302) map to carbohydrate metabolism. In BT23SHT, a locus near SWEET13 (Chr6A_601954850) corresponds to a sugar-transporter family regulating sugar partitioning in plants [33]; whether variation at this locus influences shattering in *P. juncea* remains to be tested. Moreover, the loci Chr3D_3538834 and Chr3D_3538895 annotated as cellulose synthase were identified. Cellulose synthases catalyze cellulose biosynthesis—a major cell-wall component—and thus relate to the mechanical context of seed attachment. Expression of cellulose synthase was higher in the low-shattering group than in the high-shattering group across developmental stages (Figure 7).

The *CESA* findings were contextualized by phylogeny and motif analyses. Most *CESA* proteins retained a shared set of conserved motifs, whereas species- and lineage-specific variation was also observed. Highly conserved motifs (e.g., 1, 2, 3, 6, and 12) were consistently detected, while certain motifs (e.g., 7 and 8) were absent in specific genes or species, indicating structured heterogeneity within an overall conserved framework [34,35]. These conserved motifs likely play critical roles in the core catalytic and structural functions of *CESA* enzymes, which are essential for cellulose biosynthesis. The absence of certain motifs (e.g., motifs 7 and 8) in specific species or genes suggests species-specific adaptations in cellulose biosynthesis, potentially linked to environmental adaptation, such as drought or salinity tolerance. These findings are consistent with reports that cellulose-biosynthetic genes maintain core catalytic and structural features while accommodating lineage-level divergence and environmental context [14,36,37]. In the present work, these comparative analyses situate the *P. juncea CESA* candidates within the known *CESA* landscape and provide orthogonal context for the BT23SHT chromosome 3D signal, suggesting a flexible yet conserved role of *CESA* genes in regulating seed shattering.

Several considerations emerge from these data. First, the environment specificity of associations across settings emphasizes the need to interpret single-environment signals in light of genotype-by-environment interactions. The environmental effects observed may not only reflect genetic regulation but also physiological responses to different growing conditions, such as water availability, temperature fluctuations, and soil nutrients, all of which can influence cell wall properties and seed attachment. Second, the dual-assay design (qRT-PCR and RNA-seq) supports the observed temporal trends in the abscission zone. Given the different measurement scales, interpretation is limited to within-assay comparisons rather than cross-axis numerical contrasts, and future studies should incorporate high-throughput techniques to further validate these temporal expression patterns. Third, although the loci highlighted here are supported by proximity to functionally relevant gene families and by concordant expression differences, these are observational results; functional assays are required to establish causality. Functional validation, such as gene knockouts or overexpression studies, should be conducted to determine the precise role of *CESA* genes in seed shattering.

Future Directions: (i) Functional Genetics and Histology: Manipulating *CESA* candidates (e.g., overexpression/knockout) and other cell-wall, hormone, and sugar-transport genes, coupled with abscission-zone histology, will help test their causal role in seed retention and elucidate the physical mechanisms of seed shattering. (ii) Resolution and Generality: Whole-genome resequencing and epigenomic profiling will capture low-frequency alleles and regulatory variants, while multi-environment and multi-year replication will assess the stability of associations and expression patterns. (iii) Breeding Relevance: The identified SNP markers in the 3D region, including lead SNPs, should be evaluated in broader breeding panels to assess their predictive value for seed shattering. Gene editing tools like CRISPR–Cas can validate targets for marker-assisted breeding in *P. juncea* and related species. In summary, GWAS identified a polygenic, environment-responsive architecture for seed shattering in *P. juncea*, with key loci co-localized with *CESA* candidates. These findings provide tractable targets for validation and lay the groundwork for developing molecular tools to stabilize seed retention and yield, with implications for functional genetics, breeding, and environmental adaptation.

## 4. Materials and Methods

### 4.1. Experimental Site Overview

The field experiments were conducted at two sites in Inner Mongolia, China. The Baotou experimental site is located at latitude 40°35′ N, longitude 110°34′ E, with an altitude of 1019 m. This region experiences a typical continental climate with abundant sunlight, cold and dry winters, and windy springs. The annual temperature variation is significant, with a frost-free period of approximately 140 days. The site has access to reliable irrigation, ensuring optimal growth conditions. The Hohhot experimental site is situated at latitude 40°49′ N, longitude 114°41′ E, and an altitude of 1063 m. The frost-free period ranges between 130 and 140 days. The soil at this site is sandy chestnut-calcareous, with a pH of approximately 7.6, moderate fertility, and adequate irrigation facilities.

The geographic location, soil conditions, and climatic characteristics of both experimental sites are summarized in Tables S2 and S3. These sites were chosen to represent typical growing conditions for *P. juncea* in arid and semi-arid regions, ensuring the results are representative and applicable to similar environments.

### 4.2. Experimental Materials

This study utilized 21 germplasm materials of *P. juncea* sourced from eight countries, including China, the United States, Mongolia, and Russia. Among them, one accession (CF005043) was obtained from the National Germplasm Medium-term Bank for Forage Crops in China, while the remaining 20 accessions were provided by the U.S. National Plant Germplasm System. Table S4 lists the germplasm accession numbers, countries of origin, and cultivation methods.

### 4.3. Experimental Field Design

The germplasm materials were propagated as single seedlings in a greenhouse at Inner Mongolia Agricultural University in October 2018. The growth substrate consisted of a soil and sand mixture (2:1). After establishment, the seedlings were clonally divided at the tillering nodes into two clonal groups, which were transplanted into cultivation pots. In June 2019, the two clonal groups were transferred to two experimental sites in Inner Mongolia: Hohhot and Baotou. A randomized block design was implemented at each site. For each accession, 30 individual plants were planted per site, serving as biological replicates. The spacing between plants was 50 cm, and the row spacing was 60 cm. Standard field management practices were followed during the experimental period.

### 4.4. Phenotypic Trait Measurement

For this study, 300 individual plants surviving at both experimental sites were selected (Table S4). For each plant, three inflorescences with uniform maturity and similar morphology were chosen. Each inflorescence was shaken 10 times with consistent force over a bucket with ridges, allowing detached seeds to collect in the bucket. The shaking force was standardized to ensure consistent data collection across all plants. The number of seeds shed was recorded, and the total number of seeds (shed seeds plus remaining seeds on the inflorescence) was then calculated. The seed shattering rate (SHT) was expressed as the percentage of shed seeds relative to the total seed count. The formula is as follows: SHT (%) = (number of shed seeds/total number of seeds) × 100%. The measurements were taken 30 days after full flowering when seeds had fully matured (July 2022 and 2023). The methods for assessing seed shattering in Poaceae forage crops were adapted from the standard protocol described by Larson [38]. A total of 300 individual plants were used in the study, and for each plant, three inflorescences were measured. For data consistency and reliability, three biological replicates per accession were measured. For clarity, the four experimental environments used in this study are abbreviated as follows: “HH22” and “HH23” denote the Hohhot experimental site in 2022 and 2023, respectively; “BT22” and “BT23” denote the Baotou experimental site in 2022 and 2023, respectively.

### 4.5. Genome-Wide Association Analysis

Genome-wide association analysis (GWAS) for seed shattering was performed separately for each environment using a mixed linear model (MLM) implemented in GEMMA, which controls for both kinship and population structure [39]. Because a high-quality reference genome for *P. juncea* is not yet available, the *Triticum aestivum* (wheat) genome was used for SNP alignment and annotation. Although *Hordeum vulgare* (barley) is phylogenetically closer to *P. juncea*, preliminary tests showed higher mapping rates and more complete SNP annotation with the wheat genome, so it was adopted for all downstream analyses. A total of 106,337 high-quality SNP markers were obtained from a previous genotyping-by-sequencing dataset of 300 *P. juncea* individuals [40] and filtered using the following criteria: minor allele frequency (MAF) ≥ 0.05, missing rate ≤ 10%, and retention of biallelic sites only. In the MLM, a genomic kinship matrix was calculated from the SNP data and fitted as a random effect, while population structure was accounted for by including the first principal components from a PCA of the SNP matrix as fixed covariates. GWAS was then conducted for seed shattering in each environment using this model. SNPs with *p* ≤ 1 × 10^−4^ (−log_10_(*p*) ≥ 4) were initially considered associated with seed shattering. To assess statistical reliability under multiple testing, false discovery rate (FDR) and Bonferroni-corrected *p*-values were also calculated, and SNPs that met the raw threshold and had FDR < 0.05 were treated as significant in the Results. The phenotypic variance explained (PVE) by each significant SNP was obtained from the MLM output. Manhattan and Q–Q plots were generated using the R package “CMplot”, (version 4.5.1), and candidate genes were annotated within ±100 kb of significant SNPs based on the observed LD decay pattern. Overall, the data-analysis workflow proceeded from phenotypic measurement, SNP quality control, kinship and population structure estimation, MLM-based GWAS, multiple-testing correction, and finally to candidate gene annotation.

### 4.6. Haplotype Analysis of Associated Loci

Based on the significant SNP loci identified from GWAS, haplotype analysis was conducted using HaploView [41] software to further investigate the genetic architecture of the traits.

### 4.7. Analysis of CESA Gene Expression

Samples were collected at four developmental stages (7, 14, 21, and 28 days) from the high seed shattering rate group (H group) and the low seed shattering rate group (L group). Transcriptomic analyses were conducted on the abscission zone of H group and L group [42,43]. Based on a previous analysis of the single-plant seed shattering rate in *P. juncea* [44], the average seed shattering rate in the H group and L group were 49.71% and 3.37%, respectively.

### 4.8. Clustering Analysis

To investigate the phylogenetic relationships of cellulose synthase (*CESA*) genes, this study selected 6 representative *CESA* genes from each of the species *Oryza sativa* (*LOC_Os01g54620.1*, *LOC_Os10g32980.1*, *LOC_Os03g59340.1*, *LOC_Os03g62090.1*, *LOC_Os05g08370.1*, *LOC_Os06g39970.1*), *Triticum aestivum* (*TraesCSU02G142500.1*, *TraesCS1A02G116200.1*, *TraesCS4A02G355000.1*, *TraesCS6B02G104600.1*, *TraesCS2D02G102100.1*, *TraesCS5D02G230300.1*), *Zea mays* (*Zm00001eb283110_P001*, *Zm00001eb122750_P001*, *Zm00001eb303110_P001*, *Zm00001eb306030_P001*, *Zm00001eb063800_P001*, *Zm00001eb095350_P001*), and *Arabidopsis thaliana* (*NP_195645.1*, *NP_196549.1*, *NP_001332726.1*, *NP_179768.1*, *NP_194967.1*, *NP_201279.1*), along with the *CESA* gene from *P. juncea*. Phylogenetic analysis was performed using the Neighbor-Joining method implemented in MEGA11 software [45]. The analysis was based on whole-genome sequence data, and default computational parameters were used. A bootstrap of 1000 iterations was employed to assess the stability of the tree. The phylogenetic tree revealed the evolutionary divergence and functional specialization of *CESA* genes across different species.

### 4.9. Motif Analysis

This study investigated the conservation and species-specific distribution patterns of *CESA* genes across different plants using motif analysis. The maximum number of motifs was set to 12, and MEME software was used to identify and analyze the distribution patterns of the motifs [46]. By comparing the motif distribution in *P. juncea*, *T. aestivum*, *Z. mays*, *O. sativa*, and *A. thaliana*, the study further explored the conservation of motif structures and the functional differences between species.

## 5. Conclusions

This study identifies 36 significant SNP loci associated with seed shattering in *P. juncea*, revealing its polygenic nature and environmental responsiveness. Notably, chromosome 3D harbors key loci involved in gene-environment interactions that regulate seed shattering. Motif analysis of *CESA* genes highlighted conserved and species-specific patterns, reflecting adaptation to extreme environments. Cellulose synthase genes showed stronger expression in low-shattering groups, suggesting their potential as markers for breeding stable seed retention traits. Future research should focus on functional validation using gene editing and epigenetic studies to confirm causal relationships. Additionally, integrating these findings into breeding programs via CRISPR and marker-assisted selection will enhance seed retention and improve cultivars for sustainable agriculture. These genetic insights advance our understanding of *P. juncea*’s ecological adaptation, offering applications for grassland restoration and forage crop improvement in the context of climate change.

## Figures and Tables

**Table 1:** Results of normality tests for seed shattering rates in four environments.

Variable	Test Method	Statistic	*p*-Value	Test Result
HH22SHT	Shapiro–Wilk	0.992	0.212	Following a normal distribution
	Kolmogorov–Smirnov	0.034	0.964	Following a normal distribution
BT22SHT	Shapiro–Wilk	0.976	0.0007	Deviation from normal distribution
	Kolmogorov–Smirnov	0.052	0.591	Following a normal distribution
HH23SHT	Shapiro–Wilk	0.986	0.021	Slight deviation from normal distribution
	Kolmogorov–Smirnov	0.041	0.835	Following a normal distribution
BT23SHT	Shapiro–Wilk	0.977	0.001	Deviation from normal distribution
	Kolmogorov–Smirnov	0.072	0.246	Following a normal distribution

**Table 2:** Significant SNP associated with seed shattering trait in different environments.

SNP	Chromosome	Physical Position	*p*-Value	Allele Count	MAF	R^2^ (%)	Environment
Chr2A_772842261	2A	772,842,261	2.90 × 10^−5^	C:483_T:53	0.1	4.85	HH22SHT
Chr2B_547528562	2B	547,528,562	5.12 × 10^−5^	G:568_A:32	0.05	3.53	HH22SHT
Chr2B_654317901	2B	654,317,901	4.32 × 10^−5^	C:513_T:35	0.06	5.07	HH22SHT
Chr2D_287370559	2D	287,370,559	8.54 × 10^−5^	C:415_T:87	0.17	2.38	HH22SHT
Chr2D_305918899	2D	305,918,899	9.51 × 10^−5^	G:549_T:41	0.07	1.12	HH22SHT
Chr7A_114715792	7A	114,715,792	1.25 × 10^−5^	T:454_A:64	0.12	4.93	HH22SHT
Chr7B_389130567	7B	389,130,567	4.86 × 10^−5^	C:480_T:28	0.06	7.91	HH22SHT
Chr7D_259610613	7D	259,610,613	2.56 × 10^−5^	A:475_G:41	0.08	9.59	HH22SHT
Chr1A_440143383	1A	440,143,383	2.02 × 10^−5^	C:506_T:56	0.1	7.63	BT22SHT
Chr3B_99453227	3B	99,453,227	6.17 × 10^−5^	C:447_T:39	0.08	8.91	BT22SHT
Chr4B_334746249	4B	334,746,249	7.26 × 10^−5^	C:516_T:74	0.13	8.79	BT22SHT
Chr5B_545166049	5B	545,166,049	3.40 × 10^−5^	C:454_T:38	0.08	6.17	BT22SHT
Chr5B_678529882	5B	678,529,882	2.29 × 10^−5^	G:561_A:33	0.06	7.58	BT22SHT
Chr7B_75078387	7B	75,078,387	9.05 × 10^−5^	C:429_T:159	0.27	4.8	BT22SHT
Chr7B_323723162	7B	323,723,162	7.03 × 10^−5^	C:459_T:107	0.19	7.23	BT22SHT
Chr7D_206212163	7D	206,212,163	6.87 × 10^−5^	G:482_A:34	0.07	7.15	BT22SHT
Chr1A_568332725	1A	568,332,725	1.46 × 10^−5^	A:546_G:52	0.09	8.85	HH23SHT
Chr2D_647452302	2D	647,452,302	1.98 × 10^−5^	G:489_A:101	0.17	4.31	HH23SHT
Chr4B_122827739	4B	122,827,739	4.87 × 10^−5^	G:467_A:99	0.17	6.63	HH23SHT
Chr5B_163070301	5B	163,070,301	3.12 × 10^−5^	G:405_A:89	0.18	6.99	HH23SHT
Chr6A_486007908	6A	486,007,908	7.65 × 10^−5^	T:336_C:180	0.35	4.68	HH23SHT
Chr7D_156015578	7D	156,015,578	3.72 × 10^−6^	C:428_T:152	0.26	6.66	HH23SHT
Chr7D_169986153	7D	169,986,153	5.89 × 10^−5^	C:543_T:53	0.09	6.11	HH23SHT
Chr1A_277828770	1A	277,828,770	7.72 × 10^−5^	T:354_C:236	0.4	7.16	BT23SHT
Chr1D_273648407	1D	273,648,407	1.20 × 10^−5^	G:426_A:144	0.25	6.8	BT23SHT
Chr2B_393660702	2B	393,660,702	9.11 × 10^−5^	T:398_A:120	0.23	5.37	BT23SHT
Chr3D_3538794	3D	3,538,794	6.72 × 10^−5^	C:486_T:60	0.11	7.8	BT23SHT
Chr3D_3538834	3D	3,538,834	3.26 × 10^−5^	G:502_T:56	0.1	8.44	BT23SHT
Chr3D_3538895	3D	3,538,895	1.02 × 10^−5^	T:500_C:58	0.1	9.08	BT23SHT
Chr3D_3539055	3D	3,539,055	4.18 × 10^−5^	C:500_G:58	0.1	8.01	BT23SHT
Chr3D_3539122	3D	3,539,122	3.87 × 10^−5^	T:501_A:55	0.1	8.36	BT23SHT
Chr4D_399864797	4D	399,864,797	1.64 × 10^−5^	C:488_A:56	0.1	5.66	BT23SHT
Chr4D_399865018	4D	399,865,018	2.49 × 10^−5^	G:488_A:58	0.11	5.49	BT23SHT
Chr4D_399865039	4D	399,865,039	1.30 × 10^−5^	G:490_T:56	0.1	5.74	BT23SHT
Chr6A_42883719	6A	42,883,719	8.85 × 10^−5^	G:400_A:196	0.33	5.57	BT23SHT
Chr6A_601954850	6A	601,954,850	1.03 × 10^−5^	A:494_C:26	0.05	10.58	BT23SHT

## Data Availability

Sequence data that support the findings of this study have been deposited in the Sequence Read Archive (SRA) with the primary accession code PRJNA1014568.

## Supplementary Materials

The following supporting information can be downloaded at: https://www.mdpi.com/article/10.3390/genes16111383/s1, Table S1: Annotation of candidate genes corresponding to significant SNP associated with seed shattering in environments HH22SHT, BT22SHT, HH23SHT and BT23SHT. Table S2: Environmental parameters of two locations. Table S3: Climate index of two locations. Table S4: Materials sources of *P. juncea*.
