# Supplementary material for: Genome-Wide Association Study and Candidate Gene Analysis of Seed Shattering Trait in Psathyrostachys juncea
Source: Genes (Basel). 2025 Nov 14;16(11):1383. doi: 10.3390/genes16111383 (PMC12652638; doi:10.3390/genes16111383)
Supplement: Supplementary file 1 [file genes-16-01383-s001.zip › genes-3897229-supplementary.pdf]

Supplemental Table S1. Annotation of candidate genes corresponding to significant SNP associated with seed shattering in environments HH22SHT, BT22SHT, HH23SHT and BT23SHT.

SNP	Candidate Gene	Start	End	Distance (bp)	Annotation Information	Environment
Chr2A_772842261	TraesCS2A02G580500.1	772737518	772742440	3'_99821	Disease resistance RPP13 protein	HH22SHT
Chr2B_547528562	TraesCS2B02G383800.1	547429127	547430250	5'_98312	BTB/POZ and MATH domain-containing protein	HH22SHT
Chr2B_654317901	TraesCS2B02G460000.1	654323757	654325431	5'_5856	O-methyltransferase ZRP4	HH22SHT
Chr2D_287370559	--	--	--	--	--	HH22SHT
Chr2D_305918899	TraesCS2D02G253500.1	305904462	305911538	3'_7361	WPP domain-associated protein	HH22SHT
Chr7A_114715792	--	--	--	--	--	HH22SHT
Chr7D_259610613	--	--	--	--	--	HH22SHT
Chr7B_389130567	TraesCS7B02G213300.1	389107349	389111087	5'_19480	Glyceraldehyde-3-phosphate dehydrogenase	HH22SHT
Chr1A_440143383	TraesCS1A02G248200.1	440183810	440187326	3'_40427	Rhomboid family	BT22SHT
Chr3B_99453227	TraesCS3B02G125200.1	99500619	99502567	5'_47392	1-aminocyclopropane-1-carboxylate synthase	BT22SHT
Chr4B_334746249	--	--	--	--	--	BT22SHT
Chr5B_678529882	TraesCS5B02G513600.1	678513402	678515974	3'_13908	Haloacid dehalogenase hydrolase	BT22SHT
Chr5B_545166049	--	--	--	--	--	BT22SHT
Chr7B_75078387	TraesCS7B02G069800.1	75151744	75159642	3'_73357	receptor serine/threonine protein kinase	BT22SHT
Chr7B_323723162	--	--	--	--	--	BT22SHT
Chr7D_206212163	--	--	--	--	--	BT22SHT
Chr1A_568332725	TraesCS1A02G404900.1	568265964	568267251	3'_65474	Myb DNA-binding domain	HH23SHT
Chr1A_568332725	TraesCS1A02G405600.1	568413091	568417416	5'_80366	Sugar transporter ERD6	HH23SHT
Chr2D_64	TraesCS2D02	64743002	6474328	5'_19501	F-box/LRR protein	HH23SHT

7452302	G592200.1	3	01			
Chr2D_647452302	TraesCS2D02G592500.1	647511623	647514550	3'_59321	Nucleotide hydrolase	HH23SHT
Chr4B_122827739	TraesCS4B02G110000.1	122853702	122856563	5'_25963	hypothetical protein	HH23SHT
Chr5B_163070301	TraesCS5B02G110900.1	163066563	163099320	--	Fructose-bisphosphate aldolase	HH23SHT
Chr6A_486007908	TraesCS6A02G263000.1	486011205	486011798	3'_3297	nucleolin	HH23SHT
Chr7D_156015578	TraesCS7D02G199000.1	156010919	156013075	3'_2503	Cathepsin	HH23SHT
Chr7D_169986153	TraesCS7D02G211800.1	169910706	169916257	5'_69896	K+ potassium transporter	HH23SHT
Chr3D_3538794	TraesCS3D02G010100.1	3562690	3566990	3'_23896	Cellulose synthase	BT23SHT
Chr3D_3538834	TraesCS3D02G010100.1	3562690	3566990	3'_23856	Cellulose synthase	BT23SHT
Chr3D_3538895	TraesCS3D02G010100.1	3562690	3566990	3'_23795	Cellulose synthase	BT23SHT
Chr3D_3539055	TraesCS3D02G010100.1	3562690	3566990	3'_23635	Cellulose synthase	BT23SHT
Chr3D_3539122	TraesCS3D02G010100.1	3562690	3566990	3'_23568	Cellulose synthase	BT23SHT
Chr4D_399864797	TraesCS4D02G237600.1	399777794	399779173	3'_85624	U-box domain-containing protein	BT23SHT
Chr4D_399865018	TraesCS4D02G237600.1	399777794	399779173	3'_85845	U-box domain-containing protein	BT23SHT
Chr4D_399865039	TraesCS4D02G237600.1	399777794	399779173	3'_85866	U-box domain-containing protein	BT23SHT
Chr6A_42883719	TraesCS6A02G074600.1	42922554	42927225	5'_38835	Ribosomal protein L4/L1 family	BT23SHT
Chr6A_601954850	TraesCS6A02G382500.1	6.02E+08	6.02E+08	3'_80600	Bidirectional sugar transporter SWEET13	BT23SHT
Chr1D_273648407	--	--	--	--	--	BT23SHT
Chr1A_277828770	--	--	--	--	--	BT23SHT
Chr2B_393660702	--	--	--	--	--	BT23SHT

Supplemental Table S2. Environmental parameters of two locations.

Parameter	Hohhot	Baotou
Latitude	40°49'58.3188"	40°35'54.8124"
Longitude	114°41'46.6116"	110°34'42.7512"
Altitude (m)	1063.0	1019.0
Soil pH	8.0	7.6
Total Nitrogen ($g\cdot kg^{-1}$)	1.0	1.7
Soil Organic Matter ($g\cdot kg^{-1}$)	20.7	26.6
Available Phosphorus ($mg\cdot kg^{-1}$)	20.5	6.4
Available Potassium ($mg\cdot kg^{-1}$)	171.8	205.6

Supplemental Table S3. Climate index of two locations.

Parameter	Hohhot						Baotou					
	2022			2023			2022			2023		
	May	June	July	May	June	July	May	June	July	May	June	July
Temperature (°C)	16.1	22.4	23.3	16.2	22.2	23.4	17.4	23.3	23.5	17.3	23.1	23.3
Relative Humidity (%)	30.8	41.4	52.1	38.5	33.0	52.0	35.4	45.8	60.4	44.7	41.0	62.8
Precipitation (mm)	4.7	31.3	37.2	29.7	27.3	58.5	11.0	36.7	50.0	23.9	16.3	97.4
Effective Sunshine Duration (hours)	289	246	263	226	321	241	302	281	273	253	343	269

Supplemental Table S4. Materials sources of *P. juncea.*

Accession	Individual plant number	Sample size	Longitude	Latitude	Origin	Cultivation
PI 531828	1-14	14	113°51′45″W	45°26′0″N	Idaho, U.S.	Wild
PI 595135	15-28	14	86°31′6″E	43°36′43″N	Xin jiang,China	Wild
PI 619487	29-42	14	92°4′35″E	49°51′50″N	Mongolia	Wild
CF 005043	43-56	14	110°46′″E	40°51′0″N	China	Cultivate
PI 502577	57-70	14	113°51′0″E	62°31′0″N	Russian Federation	Cultivate
PI 549118	71-84	14	111°46′0″E	40°51′0″N	Utah, U.S.	Cultivate

PI 502576	85-98	14	113°51′0″E	62°31′0″N	Russian Federation	Cultivate
PI 565060	99-112	14	112°48'30"E	63°21'2"N	Russian Federation	Wild
PI 565051	113-123	11	112°48'30"E	63°21'2"N	Russian Federation	Wild
PI 578854	124-138	15	106°7′0″W	52°2'0"N	Canada	Cultivate
PI 619483	139-153	15	90°7′58″E	49°29′13″N	Mongolia	Wild
PI 565052	154-167	14	112°50'28"E	63°50'2"N	Russian Federation	Wild
PI 531827	168-182	15	24°28′0″E	59°22′0″N	Estonia	Wild
PI 272136	183-195	13	76°55′0″E	43°19′0″N	Alma-Asa, Kazakhstan	Cultivate
PI 502573	196-210	15	164°9′5″E	65°17′0″N	Former Soviet Union	Cultivate
PI 598614	211-224	14	50°30′0″E	50°25′0″N	Kazakhstan	Wild
PI 476299	225-237	13	99°59′55″W	46°1′42″N	U.S.	Cultivate
PI 619565	238-251	14	94°55′45″E	49°22′50″N	Mongolia	Wild
PI 531826	252-268	17	93°51′45″E	42°22′6″N	China	Wild
PI 502572	269-284	16	164°9′5″E	65°17′0″N	Former Soviet Union	Cultivate
PI 598610	285-300	16	58°45′0″E	46°45′0″N	Kazakhstan	Wild
